# The American Medical Association

**Published:** 1880-07

**Authors:** 


THE AMERICAN MEDICAL ASSOCIATION.—The thirty-first annual meeting of this body last month in New York, was perhaps, taken altogether, one of the most important that has been held since its first organization. There were some really valuable papers and communications read, or presented, and a few interesting debates on subjects evolving advanced thought and earnest, careful investigation. A considerable number of the leading thinkers and workers of the profession were present and participated to a greater or less extent in the deliberations and conduct of the session. But the "small fry" were there also, and ventured their tiny fins sometimes in water far beyond the depth for which nature designed them, in their feeble efforts to move among larger and better balanced organisms. But "ever since the world began it has always been the case," and so it doubtless will be, *ad infinitum*. Professors T. Gaillard Thomas' address of welcome, was chaste, manly and appropriate. In fact just such an one as was to be expected from such a source. The President gave, notwithstanding his recent sad bereavement, an admirable address, and presented much matter worthy the deliberations of the body over which he presided.

Two admirable names were reported for the chairmanship on Practice of Medicine and Diseases of Children respectively. There were but few, if any, members of the association better suited for such positions than Professors Pepper and Jacobi. In fact, if the association hopes or cares for the good opinion of the profession at large, it must put the *practical* departments or sections of the science, into the hands and under the control, as far as possible, of gentlemen of ability and culture and not place *second* or *third* rate men upon them, merely as matters of favor or convenience to designing persons. The profession and citizens of Gotham seemed to vie with each other in rendering the sojourn of the association in their midst, as complete an ovation as possible. We would like much to speak of several things that were said and done during the sittings of the association, did space permit, and to follow the members to the theatre, and go with them upon the Messrs. Wood's grand excursion; but the pleasure was denied, and we can only add, the next meeting will be held in Richmond, in May, 1881, and express the hope that "we may be there to see."

